# Feasibility of a brief, in-patient coping and sleep intervention to reduce potentially preventable readmission of cardiac patients to hospital

**DOI:** 10.1016/j.conctc.2023.101230

**Published:** 2023-11-10

**Authors:** Helen M. Stallman, Kurt Lushington, Tamara J. Varcoe

**Affiliations:** a South Australian Medical and Health Research Institute, Adelaide, South Australia, 5000, Australia; b Thompson Institute, University of the Sunshine Coast, Birtinya, Queensland, South Australia, Australia; c Justice and Society, University of South Australia, Adelaide, South Australia, 5000, Australia

**Keywords:** Psychological distress, Anxiety, Depression, Readmission, Cardiac disorder, Inpatient

## Abstract

**Background:**

Psychological distress is prevalent amongst hospital in-patient and may predispose patients to potentially preventable readmissions after discharge. A particularly vulnerable group are patients with cardiac disorders. This study tested the feasibility of a brief cognitive behavioural therapy consisting of an in-hospital coping session and a post-discharge healthy sleep session.

**Methods:**

Standardised questionnaire were used to assess sleep, coping/distress and wellbeing at baseline (pre-intervention) and one-month post-discharge (post-intervention). Treatment fidelity and acceptability were assessed at follow-up. Participants included 72 inpatients admitted with a cardiac disorder or reported to have a cardiac problem whilst in hospital from a single Australian public hospital.

**Results:**

Most (83 %) participants found the intervention helpful/very helpful. At baseline prior to admission, almost half of participants (46 %) reported poor wellbeing, 19 % high levels of distress and poor coping, and 47 % sleeping less than 7 h per night. Following the intervention, 45 % of participants with poor wellbeing at baseline had reliable change in wellbeing at follow-up. Conversely, only 22 % of patients with high levels of coping/distress at baseline demonstrated improved coping/distress at follow-up suggesting smaller gains. On average a large 43 min gain in sleep duration was observed post-treatment in patients with poor sleep at baseline. Fourteen percent of participants were readmitted to hospital within 34-days of discharge.

**Conclusions:**

The coping and sleep intervention was well received with positive outcomes in patients especially those reporting high levels of distress for sleep and to lesser extent coping and wellbeing. Future studies to assess the efficacy of the brief intervention at reducing hospital readmissions are needed.

## 1 Introduction

Hospital readmission rates are an important indicator of care [1,2] and account for a significant proportion of hospital expenditure [3]. In Australia, for example, preventable hospital readmissions contribute to 6.6 % of all hospitalisations [4]. Readmissions are considered to be clinically related to a prior admission and therefore potentially preventable, " … if there was a reasonable expectation that it could have been prevented by one or more of the following: 1) the provision of quality care in the initial hospitalisation, 2) adequate discharge planning, 3) adequate post-discharge follow-up, or 4) improved coordination between in-patient and outpatient health care teams” [5, p. 76]. Preventable readmissions are associated with service delivery factors including incomplete treatment, inadequate treatment of the underlying problem, poor coordination of services at the time of discharge and inadequate access to care [6,7]. They are also associated with individual factors and, in particular, psychological distress at discharge [[8], [9], [10]]. Prominent among those vulnerable to psychological distress are patients with cardiac disorders [[11], [12], [13]]. Patients with cardiac disorders also have a high rate of readmission varying up to 23 % for percutaneous coronary intervention and up to 57 % for heart failure [14,15]. In patients with cardiac disorders, psychological interventions are reported to increase treatment adherence [[16], [17], [18], [19]] and reduce preventable hospital readmissions [9,20,21]. However apart for nurse-led programs providing in-hospital education (e.g., the disease process, early symptom recognition, self-care strategies, medication management, nutrition, and lifestyle modification) [22], trials specifically testing psychological interventions have been limited and where programs have been implemented they have been delivered post-discharge [[23], [24], [25]]. Early intervention is recommended for most psychology problems [26]. This raises the possibility that including an intervention arm while patients are in hospital may allow for further gains, but the feasibility of an in-hospital program remains to be tested.

Psychological distress is caused by problems in any one or more of the biopsychosocial determinants of health and wellbeing: healthy environments, developmental competencies (healthy identity, emotional and behavioural regulation, interpersonal skills, problem-solving skills), sense of belonging, health behaviours (sleep, nutrition, exercise), coping, resilience (perception of innate resilience) and illness (physical and psychological) [27,28]. The two proximal domains to high levels of distress—sleep and coping—may be potentially helpful intervention targets to improve mood regulation and consequently improve treatment adherence and health outcomes in patients with cardiac disorders. Patients with cardiac disorders typically report poor sleep [9,[29], [30], [31], [32]] and problems with psychological adjustment, i.e. coping [[33], [34], [35]]. They also respond well to cognitive behavioural programs delivered post-discharge at improving sleep [36,37] and coping [38,39]. Validated brief interventions have been developed for sleep hygiene [40,41] and coping [27,42], which could be adapted for in-patient delivery in patients with cardiac disorders.

In summary, the primary aim of this study was to assess the feasibility of a brief two-session (1 *x* in-hospital and 1 *x* post-discharge) psychological intervention to improve wellbeing, coping and sleep and, thereby, reduce distress and preventable hospital readmissions. The secondary aim was to examine the impact of the psychological intervention on wellbeing, coping, self-efficacy, self-reported sleep quality/quality and help seeking intentions.

## 2 Materials and method

### 2.1 Study design

This study used a pre-test post-test design with a 1-month follow-up.

### 2.2 Participants

Participants were 72 inpatients admitted for a cardiac disorder or were reported to have cardiac problems whilst in hospital secondary to other medical issues. Patients were recruited from three wards in an Australian public hospital. Inclusion criteria were 18 years or older, medically stable, and able to provide informed consent and participate. Exclusion criteria were mental status impairment and poor English language skills.

### 2.3 Measures

**Demographics.** Demographics collected were date of birth, sex, and whom the patient lived with (spouse or partner; family members; friends; nursing home; alone; other).

The primary outcomes are feasibility and acceptability of the intervention, explored through rate of recruitment, intervention implementation, treatment fidelity, readmissions, retention and perceived usefulness of the intervention. Acceptability was assessed with a question about use and how helpful the intervention was, and whether participants would recommend it to other patients. The treatment-use questions were: 1) Did you use your coping plan? And if so, 2) was the plan effective; and 3) Did you edit your plan?; and 4) Did you do anything differently after the sleep intervention?

**Treatment fidelity.** The project coordinator completed treatment integrity checklists after each treatment component using treatment integrity checklists—10 items for Care Collaborate Connect and eight items for the sleep intervention. Intervention steps were rated as not done (0), attempted, but some elements were missing (1) or delivered competently (2). The total competency scores ranged from 0 to 20 for Care Collaborate Connect and 0 to 16 for the sleep intervention.

Reliability of ratings of the need for additional professional support to cope in Care Collaborate Connect part of the intervention was compared against needs determined from self-reported unhealthy coping subscale of the Coping Index.

The secondary outcomes were wellbeing, coping, coping self-efficacy, self-reported sleep quality and quantity and help-seeking intentions.

***Wellbeing.*** The WHO-5 [43], a 5-item measure of subjective wellbeing, was used as it is not susceptible to the reliability and validity limitations of negative psychological states, such as psychological distress [44,45]. Responses are rated on a 6-point Likert-type scale, all of the time (5), most of the time (4), more than half of the time (3), less than half of the time (2), some of the time (1), and at no time (0). The total score ranges from 0 to 25, with scores lower than 13 indicating poor wellbeing. The WHO-5 had excellent internal consistency in this sample, α = 0.90.

***Coping/distress***. The Coping Index (CI) [46] is a 20-item measure of healthy and unhealthy coping strategies. The measure is based on the Health Theory of Coping [27]. Each item is rated on a 4-point Likert-type scale: I don't do this at all (0), I do this occasionally (1), I do this often (2), and I do this most of the time (3). The healthy and unhealthy subscale scores are the average of the ten scale item scores and range from 0 to 3. Unhealthy scores greater than 0.75 are excellent predictors of distress indicative of psychiatric illness and need for additional professional support [47] and this criterion was used to identify ‘high’ distress participants.

***Coping self-efficacy.*** The Coping Self-Efficacy Index (CCSEI) [48] is a 5-item measure of confidence to use healthy coping strategies when distressed. Each item is rated on a 4-point scale from 0 (not confident at all) to 3 (very confident). The total score is the sum of the items, with higher scores indicating better coping self-efficacy. Total scores range from 0 to 15, with higher scores indicating better wellbeing. The CSEI has adequate internal consistency in this sample, Cronbach α = 0.77.

***Help-seeking intentions*.** The Help-Seeking Intentions Scale (HSIS) [49] is a 7-item measure of how likely participants would seek support from a range of social and professional supports if they were distressed and had difficulty coping in the future, rated on a scale from 0 (not likely at all) to 6 (very likely). The scale was author constructed and demonstrated excellent internal consistency, Cronbach α = 0.81.

***Sleep****:* Subjective sleep quantity and quality were assessed through a series of questions asking the participant to reflect on typical sleep at home. Questions were average hours of sleep, sleep quality, daytime sleepiness, sleep onset delay, night awakenings, and the impact of poor sleep on mood, concentration and relationships.

### 2.4 Procedure

Ethics was approved by the Central Adelaide Local Health Network Human Research Ethics Committee (number 595). In accordance with the tenets of the Declaration of Helsinki, all participants provided written informed consent for study participation. No stipend was provided to participants. Participants were recruited between February to October 2020. Eligible patients were identified by medical or nursing staff on each ward. Nursing staff obtained permission from eligible patients for the project coordinator to meet with them to discuss the study. The study was described to patients as a study about patient wellbeing and coping. Participants had the option to complete baseline assessment on paper or online. After the baseline assessment, the project coordinator delivered the Care Collaborate Connect intervention (coping intervention) to the patient at their bedside. The sleep intervention was delivered via telephone one-week post-discharge. A mood and coping review were done before the sleep intervention. Follow-up assessments were completed 1-month post-discharge online with the URL emailed to participants (*n* = 54), on a paper questionnaire mailed to a participant (*n* = 4), or over the telephone (*n* = 12); two withdrew before follow-up. Participants were emailed or mailed up to three times to complete a follow-up assessment. The project coordinator completed Care Collaborate Connect: Suicide Prevention training [42] and was supervised a Clinical Psychologist. The project coordinator completed the self-report treatment integrity checklists after each intervention.

### 2.5 Intervention

**Coping**.Care Collaborate Connect is a low-intensity psychological intervention that normalises distress and supports healthy coping [27,42]. The intervention involves providing psychoeducation about coping, reviewing patients' current healthy and unhealthy coping strategies, and supporting patients to strengthen their coping plan with additional professional supports if needed. Participants are encouraged to record their healthy coping strategies on a My Coping Plan worksheet or the My Coping Plan app [50]. Participants and the project coordinator collaboratively decide if the participant has high needs for additional professional support (immediate support), moderate needs (may need ad hoc professional support), or low needs (has few or no unhealthy coping strategies or adequate social and/or professional support).

A mood and coping review were conducted at subsequent sessions. If participants experienced any unpleasant emotions since the coping intervention, they are asked how they coped. A review of the effectiveness of their coping plan follows, and participants are encouraged to strengthen their plan by adding additional supports if it was not effective. Self-directed Coping Planning has been shown to significantly reduce short-term psychological distress and improve wellbeing [45].

**Sleep.** Sleep hygiene is an effective strategy to reduce daytime sleepiness and improve sleep quality [51,52]. The sleep session involved a review of sleep hygiene behaviours based on previous studies that have used cognitive behaviour for insomnia in patients with cardiac disorders [36,37]. Participants who had sleep problems were encouraged to identify and set goals for improving sleep hygiene behaviours. As hospital environments disrupt patient sleep [53], the sleep intervention was conducted one-week post-discharge when patients returned to normal routines.

### 2.6 Statistical analysis

Descriptive statistics were used to describe the sample and recruitment, retention, and data completion rates. Completer and non-completers were compared on outcomes variables using Pearson Chi-square tests for categorical variables or independent samples *t*-tests for continuous variables. Multiple imputation was used on missing data for outcome variables. The Reliable Change Index [54] was used to assess reliable change on measures from baseline to one-month follow-up. Statistical tests were two-sided with a *p* < .05 significance level.

## 3 Results

### 3.1 Recruitment

Recruitment was conducted on weekdays for six months. One hundred and seventy-eight patients were approached by nursing staff, of which 145 (81 %) consented to learn more about the study, and 72 (40 %) agreed to participate. Reasons for non-participation included: not thinking the study was relevant to them, being too tired or unwell, being unable to commit to follow-up, or being too worried about their health to concentrate. Almost all participants (97 %) completed the baseline questionnaire on paper rather than online. Twenty-two per cent of participants did not have an email address for communication, so mail was used for post-discharge appointment setting and assessments.

### 3.2 Sample characteristics

The average age of participants was 64.4 years (*SD* = 11.3 years; Range = 35–86). Most participants were male (61 %; 44/72) and lived with a partner (61 %; 44/72); 13 % (9/72) lived with other family members, 1 % lived with friends (1/72) and 25 % (18/72) lived alone. Participants were from the cardiology (74 %, 53/72), respiratory (8 %, 6/72) or surgical wards (18 %, 13/72). The average number of days hospitalised was 6.0 (*SD* = 7.0; Range = 1–38). Participants were treated for one or more of the following cardiac conditions: 54 % (39/72) for coronary heart disease, 13 % (9/72) heart arrhythmia, 6 % (4/72) heart valve disease, 3 % (2/72) heart failure, 2 % (1/72) hypertension, 2 % (1/72) myocarditis, and 25 % (18/72) other (cardiac event post-surgery or secondary to other medical condition).

At baseline, 68 % of the sample had a least one problem associated with mood dysregulation (poor wellbeing, high distress indicated by unhealthy coping or sleep problems). This includes 46 % with poor wellbeing, 19 % with high distress, and 15 % with poor wellbeing and high distress. Two participants had poor wellbeing, high distress, and sleep problems. At 1-week post-discharge, 39 % reported sleep problems. Of those with sleep problems, 5 % had onset insomnia problems, 12 % had maintenance insomnia problems, 3 % had both onset and maintenance insomnia problems, 12 % woke to feel unrefreshed, and 8 % had insomnia and woke to feel unrefreshed.

### 3.3 Intervention implementation

The average duration to deliver the coping intervention was 37.5 min (*SD* = 10.5; Range = 20–71). Most participants (76 %) had low needs for additional professional support to cope, 24 % had moderate needs for additional professional support, and none had high needs for additional professional support. All participants with moderate needs identified suitable professional supports and included them in their coping plan.

Almost all (92 %) participants attended the sleep session one week following discharge. Only one participant declined the intervention, three were unable to be contacted, one was readmitted to another hospital and was lost to the study, and one withdrew from the study. Of those who reported sleep problems (39 % of participants), most (62 %) developed a plan to improve their sleep hygiene. The average duration of the sleep intervention was 15.5 min (*SD* = 5.3 min, range = 7–27 min).

### 3.4 Treatment fidelity

The average treatment fidelity score was very high for both the coping intervention (*M* = 18.7, *SD* = 1.9) and the sleep interventions (*M* = 15.2, *SD* = 1.1). Of the 14 participants who self-reported unhealthy coping strategies indicative of high distress at baseline, 71 % (*n* = 10) were identified as needing additional professional support during the intervention. Of the participants who self-reported high distress but were assessed as having low needs for additional professional support to cope during the intervention, one participant already had regular counselling. Three additional participants reported high distress in the self-report questionnaires, but this was not identified in the intervention.

### 3.5 Retention

Completion of the two intervention components was very high (92 %), however, there was a 26 % attrition rate by one-month follow-up assessment. Of those who were lost to follow-up, 68 % had poor wellbeing and 26 % had high distress at baseline. Two participants withdrew from the study, and 23.6 % were unable to be contacted at follow-up. Compared with non-completers, those who completed the follow-up assessment had a shorter in-patient stays (*t* (69) = 3.08, *p* = .003), better wellbeing (*t* (70) = 2.40, *p* = .02) and coping self-efficacy at baseline (*t* (70) = 2.12, *p* = .04). There was no significant difference between the groups on unhealthy (*t* (70) = 0.05, *p* = .96) or healthy coping (*t* (70) = 0.40, *p* = .69).

### 3.6 Perceived usefulness of the intervention

Of those who experienced unpleasant emotions during the 1-week following discharge, 64 % (*n* = 7) of participants reported using their Coping Plan. Almost half (43 %) found their plan effective (*n* = 3) and 57 % edited the plan to make it more effective (*n* = 4). There was no significant effect of baseline wellbeing (χ^2^ (1, *N* = 11) = 0.51, *p =* .48), or high distress (χ^2^ (1, *N* = 11) = 1.64, *p* = .20), on the proportion of participants who used their coping plan within one-week.

At one-month follow-up, 21 % of participants found the coping intervention helpful or somewhat helpful (62 %); 7 % did not find it helpful at all. There was no significant effect of baseline wellbeing (χ^2^ (1, *N* = 64) = 0.78, *p =* .37) or high distress (χ^2^ (1, *N* = 64) = 1.01, *p* = .32) on the proportion of participants who found the coping intervention helpful.

At one-month follow-up, 14 % of all participants reported the sleep intervention was helpful, and 71 % found it somewhat helpful; 15 % did not find it helpful at all. Of those with sleep problems at baseline, 37 % of participants changed their behaviour post-intervention. There was no significant difference between participants with and without sleep problems on the perception of the usefulness of the sleep intervention (χ^2^ (2, *N* = 51) = 2.25, *p =* .33) or behaviour change (χ^2^ (1, *N* = 50) = 0.69, *p* = .41). Most participants (92 %) would recommend the coping and sleep intervention for other patients.

### 3.7 Intervention outcomes

Table 1 shows the means and standard deviations at baseline and 1-month follow-up for participants who had poor wellbeing and high distress at baseline. Overall, 45 % of participants with poor wellbeing and 22.2 % of participants with high distress had reliable change in wellbeing at follow-up. By contrast, only a few of the participants with poor wellbeing demonstrated improvement in unhealthy coping strategies (10 %). However, one-fifth of those with unhealthy coping strategies showed reliable change at follow-up. This suggests that high distress patients were reasonably responsive to intervention. Fifteen per cent of participants with poor wellbeing showed improvements in coping self-efficacy compared with none of those with high distress. Finally, none of the participants with either poor wellbeing or high distress showed reliable improvements in help-seeking intentions. For the poor wellbeing group, mean HSI scores at baseline were 17.75 (*SD* = 5.94) and at one-month follow up were 18.95 (*SD* = 8.93). For the high distress group, mean HSI scores at baseline were 19.88 (*SD* = 6.41) and at one-month follow up were 20.22 (*SD* = 4.38).

**Table 1:** Baseline and 1-month follow-up mean, standard deviations, and proportion of sample with reliable change on outcomes variables for the participants with poor wellbeing and high distress at baseline.

*Variable*	Poor wellbeing *n* = 20			High distress *n* = 9		
	*M* (*SD*)		% RCI	*M* (*SD*)		*%* RCI
	Baseline	1-month follow-up		Baseline	1-month follow-up	
WHO-5	8.60 (3.05)	13.45 (4.99)	45.0	10.89 (3.95)	13.67 (5.41)	22.2
Coping – Healthy	1.14 (0.39)	1.27 (0.35)	15.0	1.07 (0.36)	1.24 (0.34)	22.2
Coping – Unhealthy	0.68 (0.32)	0.58 (0.31)	10.0	1.00 (0.17)	0.81 (0.34)	22.2
CSEI	9.90 (2.22)	10.90 (2.08)	15.0	10.00 (2.18)	10.22 (1.89)	0

Note: CSEI = Coping Self Efficacy Index, RCI = Reliable Change Index, % RCI = proportion of participants that showed significant improvements. And WHO-5 = World Health Organisation-5.

The average sleep duration for the sample was just below the lower recommended limit at baseline (*M* = 6.82 h, *SD =* 1.84) with 47 % of participants reporting fewer than 7 h of sleep a night. By follow-up, the average sleep duration was 7.00 h (*SD =* 1.68). For participants who had fewer than 7 h of sleep at baseline, 8.3 % had positive, reliable improvements at follow-up with their mean sleep duration increasing from 5.27 h (*SD* = 1.14) to 5.99 h (1.31).

### 3.8 Readmissions

A readmission was defined as an admission happening more than 24 h after the index admission. The 24 h criteria was chosen to limit ambiguous cases and improve data clarity (e.g., a readmission within 24 h might be considered a continuation of the initial hospital stay rather than a distinct and separate event), to remain consistent with other studies that have used the same criteria and the impracticality of providing the intervention to same day patients. Notably and notwithstanding the 24 h criteria, the earliest readmission in those participants not lost to follow-up was 14 days post-discharge. Fourteen percent (10/72) of participants were readmitted to hospital approximately within one-month of discharge (14–34 days). Apart for one patient who was readmitted to another hospital and follow-up details are incomplete, the remaining readmissions were for cardiac-related problems. Of the remaining readmissions, 66 % percent (6/9) were previously admitted for coronary heart disease, 22 % (2/9) for heart arrhythmia and 22 % (2/9) for other cardiac-related problems. All readmissions were unplanned. There was no significant difference between those who were or were not readmitted to hospital on age (*t* (51) = 1.14, *p* = .26), sex (χ^2^ (df 1, *N* = 53) = 0.35, *p =* .55), in-patient days (*t* (51) = 0.39, *p* = .70), whether they were living alone or with someone else (χ^2^ (df 1, *N* = 53) = 0.01, *p =* .97), use of coping plan (χ^2^ (df 1, *N* = 52) = 1.60, *p =* .45), baseline wellbeing (*t* (51) = 0.24, *p* = .81) or baseline high distress (*t* (51) = 1.08, *p* = .29).

## 4 Discussion

This study tested the feasibility of a brief sleep and coping intervention that included an in-hospital components to reduce potentially preventable hospital readmissions secondary to psychological distress. Despite expectations, 14 % of participants were readmitted to hospital within approximately one month of discharge—a rate consistent with the current 30-day post-discharge rate of readmissions in South Australian public hospitals (21 %) [55] and the Australian readmissions for cardiac patients (6.3–27 %) [2]. Moreover, readmission rates were not predicted by either demographics or outcomes measures. However, the intervention received high acceptance and in the patients with high levels of distress, promising, but modest outcome gains.

Of the participants recruited, fewer than half of participants had poor wellbeing, and only one fifth had unhealthy coping scores indicative of high distress. These rates are similar to previous reports of psychological distress in in-patient populations [20,56], unhealthy coping indicative of mental illness [57] and psychiatric illness in the general population [58]. Nevertheless, and regardless of severity, almost all participants found the brief intervention helpful or very helpful. In addition, the retention rate was high, and most participants reported that they would recommend the intervention to others. Finally, consistent with evidence that additional assessment and intervention is of benefit, in the subset of cardiac patients with high distress the brief intervention produced reliable changes at follow-up [28,47].

The delivery of an in-hospital intervention is not without challenges. The learnings from the present study include the importance of scheduling and staff engagement. Fewer than half of the patients approached consented to participate because of short hospital stays, crowded treatment regimens and other timing issues. Where possible pre-screening of mood and coping may assist [59]. Recruitment was dependent on staff availability and promotion. In future, greater gains may be achieved by adapting the screening process to fit within the standard organisational procedures rather than as an additional task. For example and consistent with recent Australian governmental productivity and mental health review recommendations [60,61]. The latter could be achieved by including a routine mood and coping review whilst patients are in-hospital to identify those at risk. Finally, gains were not observed in health seeking intentions for psychological problems. Mental health literacy programs are reported to alter help-seeking attitudes but there is less evidence that they modify help-seeking behaviour [62].

This study has several limitations. First, the limited uptake of the intervention by patients and loss of patients at follow-up. Those who did compete the program tended to have shorter in-patient stays and better wellbeing and coping self-efficacy at baseline. This may have influenced the sample representativeness. Second, the limited sample size prevented sub-analysis and it is possible that the efficacy of in-hospital psychological interventions may differ according to cardiac disorder. Third, the intervention was not compared to a concurrently recruited control condition or matched controls, thus limiting conclusions regarding the effectiveness of the psychological intervention. Building on the findings from the present feasibility study, these limitations can be addressed in future research.

## 5 Conclusions

This study examined the feasibility of a brief coping and sleep intervention for inpatients with a history of cardiac disorders. The intervention had a high degree of acceptability and demonstrated gains in patients with poor wellbeing and/or high levels of psychological distress especially for sleep and to a lesser extent coping and wellbeing. Despite expectations, hospital readmissions were not reduced. However, it is acknowledged that efficacy studies will be needed to evaluate more fully the Care Collaborate Connect and sleep intervention model. The present findings point to the utility of modifying clinical practice in hospitals to address the biopsychosocial needs of vulnerable patients.

## Funding

This study was funded by the 10.13039/100009727Hospital Research Foundation.

## Author contributions

HS, KL and TV contributed to study design and manuscript preparation. HS and TV contributed to recruitment, data collection and analyses.

## Declaration of competing interest

The authors declare that they have no known competing financial interests or personal relationships that could have appeared to influence the work reported in this paper.

## Data Availability

The authors do not have permission to share data.

## References

[1] Friedman B., Basu J. (2004). The rate and cost of hospital readmissions for preventable conditions. Med. Care Res. Rev..

[2] Labrosciano C., Air T., Tavella R., Beltrame J.F., Ranasinghe I. (2020). Readmissions following hospitalisations for cardiovascular disease: a scoping review of the Australian literature. Aust. Health Rev. : a publication of the Australian Hospital Association.

[3] Anderson G.F., Steinberg E.P. (1984). Hospital readmissions in the medicare population. N. Engl. J. Med..

[4] Australian Institute of Health Welfare (2019).

[5] Goldfield N.I., McCullough E.C., Hughes J.S., Tang A.M., Eastman B., Rawlins L.K., Averill R.F. (2008). Identifying potentially preventable readmissions. Health Care Financ. Rev..

[6] Halfon P., Eggli Y., Pêtre-Rohrbach I., Meylan D., Marazzi A., Burnand B. (2006). Validation of the potentially avoidable hospital readmission rate as a routine indicator of the quality of hospital care. Med. Care.

[7] Kripalani S., Lefevre F., Phillips C.O., Williams M.V., Basaviah P., Baker D.W. (2007). Deficits in communication and information transfer between hospital-based and primary care physicians. JAMA.

[8] Tully P.J., Baker R.A., Turnbull D., Winefield H. (2008). The role of depression and anxiety symptoms in hospital readmissions after cardiac surgery. J. Behav. Med..

[9] Rutledge T., Reis V.A., Linke S.E., Greenberg B.H., Mills P.J. (2006). Depression in heart failure a meta-analytic review of prevalence, intervention effects, and associations with clinical outcomes. J. Am. Coll. Cardiol..

[10] Vámosi M., Lauberg A., Borregaard B., Christensen A.V., Thrysoee L., Rasmussen T.B., Ekholm O., Juel K., Berg S.K. (2020). Patient-reported outcomes predict high readmission rates among patients with cardiac diagnoses. Findings from the DenHeart study. Int. J. Cardiol..

[11] De Hert M., Detraux J., Vancampfort D. (2018). The intriguing relationship between coronary heart disease and mental disorders. Dialogues Clin. Neurosci..

[12] Correa-Rodríguez M., Abu Ejheisheh M., Suleiman-Martos N., Membrive-Jiménez M.J., Velando-Soriano A., Schmidt-RioValle J., Gómez-Urquiza J.L. (2020). Prevalence of depression in coronary artery bypass surgery: a systematic review and meta-analysis. J. Clin. Med..

[13] Cohen B.E., Edmondson D., Kronish I.M. (2015). State of the art review: depression, stress, anxiety, and cardiovascular disease. Am. J. Hypertens..

[14] Bottle A., Honeyford K., Chowdhury F., Bell D., Aylin P. (2018). Factors associated with hospital emergency readmission and mortality rates in patients with heart failure or chronic obstructive pulmonary disease. a national observational study.

[15] Korda R.J., Du W., Day C., Page K., Macdonald P.S., Banks E. (2017). Variation in readmission and mortality following hospitalisation with a diagnosis of heart failure: prospective cohort study using linked data. BMC Health Serv. Res..

[16] Dimatteo M.R., Lepper H.S., Croghan T.W. (2000). Depression is a risk factor for noncompliance with medical treatment. Arch. Intern. Med..

[17] Eisele M., Harder M., Rakebrandt A., Boczor S., Marx G., Blozik E., Trader J.M., Stork S., Herrmann-Lingen C., Scherer M. (2020). Association of depression and anxiety with adherence in primary care patients with heart failure-cross-sectional results of the observational RECODE-HF cohort study. Fam. Pract..

[18] Crawshaw J., Auyeung V., Norton S., Weinman J. (2016). Identifying psychosocial predictors of medication non-adherence following acute coronary syndrome: a systematic review and meta-analysis. J. Psychosom. Res..

[19] Ziegelstein R.C., Fauerbach J.A., Stevens S.S., Romanelli J., Richter D.P., Bush D.E. (2000). Patients with depression are less likely to follow recommendations to reduce cardiac risk during recovery from a myocardial infarction. Arch. Intern. Med..

[20] Reuter K., Raugust S., Marschner N., Härter M. (2007). Differences in prevalence rates of psychological distress and mental disorders in inpatients and outpatients with breast and gynaecological cancer. Eur. J. Cancer Care.

[21] Cossette S., Frasure-Smith N., Lespérance F. (2001). Clinical implications of a reduction in psychological distress on cardiac prognosis in patients participating in a psychosocial pntervention program. Psychosom. Med..

[22] Rizzuto N., Charles G., Knobf M.T. (2022). Decreasing 30-day readmission rates in patients with heart failure. Crit. Care Nurse.

[23] Whalley B., Thompson D.R., Taylor R.S. (2014). Psychological interventions for coronary heart disease: cochrane systematic review and meta-analysis. Int. J. Behav. Med..

[24] Huffman J.C., Millstein R.A., Mastromauro C.A., Moore S.V., Celano C.M., Bedoya C.A., Suarez L., Boehm J.K., Januzzi J.L. (2016). A positive psychology intervention for patients with an acute coronary syndrome: treatment development and proof-of-concept trial. J. Happiness Stud..

[25] Zambrano J., Celano C.M., Januzzi J.L., Massey C.N., Chung W.J., Millstein R.A., Huffman J.C. (2020). Psychiatric and psychological interventions for depression in patients with heart disease: a scoping review. J. Am. Heart Assoc..

[26] Arvidsdotter T., Marklund B., Kylén S., Taft C., Ekman I. (2016). Understanding persons with psychological distress in primary health care. Scand. J. Caring Sci..

[27] Stallman H.M. (2018). Coping planning: a patient-centred and strengths-focused approach to suicide prevention training. Australas. Psychiatr..

[28] Stallman H.M. (2020). Suicide following hospitalisation: systemic treatment failure needs to be the focus rather than risk factors. Lancet Psychiatr..

[29] Parish J.M. (2009). Sleep-related problems in common medical conditions. Chest.

[30] Rao A. (2005). Impact of heart failure on quality of sleep. Postgrad. Med..

[31] Redeker N.S., Hedges C. (2002). Sleep during hospitalization and recovery after cardiac surgery. J. Cardiovasc. Nurs..

[32] Javaheri S., Redline S. (2017). Insomnia and risk of cardiovascular disease. Chest.

[33] Steptoe A., Kivimäki M. (2012). Stress and cardiovascular disease. Nat. Rev. Cardiol..

[34] Livneh H. (1999). Psychosocial adaptation to heart diseases: the role of coping strategies. J. Rehabil..

[35] Kubzansky L.D., Huffman J.C., Boehm J.K., Hernandez R., Kim E.S., Koga H.K., Feig E.H., Lloyd-Jones D.M., Seligman M.E.P., Labarthe D.R. (2018). Positive psychological well-being and cardiovascular disease. J. Am. Coll. Cardiol..

[36] Heenan A., Pipe A., Lemay K., Davidson J.R., Tulloch H. (2020). Cognitive-behavioral therapy for insomnia tailored to patients with cardiovascular disease: a pre–post study. Behav. Sleep Med..

[37] Siebmanns S., Johansson P., Ulander M., Johansson L., Andersson G., Broström A. (2021). The effect of nurse-led Internet-based cognitive behavioural therapy for insomnia on patients with cardiovascular disease: a randomized controlled trial with 6-month follow-up. Nursing Open.

[38] Linden W., Phillips M.J., Leclerc J. (2007). Psychological treatment of cardiac patients: a meta-analysis. Eur. Heart J..

[39] Magán I., Jurado-Barba R., Casado L., Barnum H., Jeon A., Hernandez A.V., Bueno H. (2022). Efficacy of psychological interventions on clinical outcomes of coronary artery disease: systematic review and meta-analysis. J. Psychosom. Res..

[40] Irish L.A., Kline C.E., Gunn H.E., Buysse D.J., Hall M.H. (2015). The role of sleep hygiene in promoting public health: a review of empirical evidence. Sleep Med. Rev..

[41] Kamal M., Herawati T. (2019). The effect of sleep hygiene and relaxation Benson on improving the quality of sleep among health failure patients: a literature review. IJNHS.

[42] Stallman H.M. (2019).

[43] (1998). Psychiatric Research Unit Frederiksborg General Hospital, WHO (Five) Well-Being Index.

[44] Shrout P.E., Stadler G., Lane S.P., McClure M.J., Jackson G.L., Clavél F.D., Iida M., Gleason M.E.J., Xu J.H., Bolger N. (2018). Initial elevation bias in subjective reports. Proc. Natl. Acad. Sci. USA.

[45] Stallman H.M. (2019). Efficacy of the My Coping Plan mobile application in reducing distress: a randomised controlled trial. Clin. Psychol..

[46] Stallman H.M. (2017).

[47] Stallman H.M., Beaudequin D., Hermens D.F., Eisenberg D. (2021). Modelling the relationship between healthy and unhealthy coping strategies to understand overwhelming distress: a Bayesian network approach. Journal of Affective Disorders Reports.

[48] Stallman H.M. (2018).

[49] Stallman H.M. (2019).

[50] Stallman H.M. (2017).

[51] Kakinuma M., Takahashi M., Kato N., Aratake Y., Watanabe M., Ishikawa Y., Kojima R., Shibaoka M., Tanaka K. (2010). Effect of brief sleep hygiene education for workers of an information technology company. Ind. Health.

[52] Hershner S., O'Brien L.M. (2018). The impact of a randomized sleep education intervention for college students. J. Clin. Sleep Med..

[53] Dobing S., Frolova N., McAlister F., Ringrose J. (2016). Sleep quality and factors influencing self-reported sleep duration and quality in the general internal medicine inpatient population. PLoS One.

[54] Jacobson N.S., Truax P. (1991). Clinical significance: a statistical approach to defining meaningful change in psychotherapy research. J. Consult. Clin. Psychol..

[55] Sharma Y., Horwood C., Hakendorf P., Au J., Thompson C. (2020). Characteristics and clinical outcomes of index versus non-index hospital readmissions in Australian hospitals: a cohort study. Aust. Health Rev. : a publication of the Australian Hospital Association.

[56] Mitchell A.J., Chan M., Bhatti H., Halton M., Grassi L., Johansen C., Meader N. (2011). Prevalence of depression, anxiety, and adjustment disorder in oncological, haematological, and palliative-care settings: a meta-analysis of 94 interview-based studies. Lancet Oncol..

[57] Stallman H.M., Eisenberg D. (2020).

[58] Slade T., Johnston A., Oakley Browne M.A., Andrews G., Whiteford H. (2007). National survey of mental health and wellbeing: methods and key findings. Aust. N. Z. J. Psychiatr..

[59] McCaffrey R., Taylor N. (2005). Effective anxiety treatment prior to diagnostic cardiac catheterization. Holist. Nurs. Pract..

[60] National Mental Health Commission (2019).

[61] Productivity Commission (2020). Mental Health, Report.

[62] Gulliver A., Griffiths K.M., Christensen H., Brewer J.L. (2012). A systematic review of help-seeking interventions for depression, anxiety and general psychological distress. BMC Psychiatr..

